# In Vitro Characterization of Human CD24^hi^CD38^hi^ Regulatory B Cells Shows CD9 Is Not a Stable Breg Cell Marker

**DOI:** 10.3390/ijms22094583

**Published:** 2021-04-27

**Authors:** Fatin N. Mohd Jaya, Sergio G. Garcia, Francesc E. Borras, Dolores Guerrero, Godfrey C. F. Chan, Marcella Franquesa

**Affiliations:** 1 School of Biomedical Sciences, Faculty of Medicine, The University of Hong Kong, 21 Sassoon Road, Hong Kong, China; fatinjay@hku.hk; 2 REMAR-IVECAT Group, Health Science Research Institute Germans Trias i Pujol, Can Ruti Campus, 08916 Badalona, Spain; sggarcia@igtp.cat (S.G.G.); feborras@igtp.cat (F.E.B.); 3 Otorhinolaryngology Department, Hospital Universitari Germans Trias i Pujol, 08916 Badalona, Spain; mdguerrero.germanstrias@gencat.cat; 4 Department of Pediatrics and Adolescent Medicine, Faculty of Medicine, The University of Hong Kong, 21 Sassoon Road, Hong Kong, China; gcfchan@hku.hk

**Keywords:** CD9, regulatory B cells, immune tolerance, immune homeostasis, inflammation

## Abstract

Regulatory B (Breg) cells are endowed with immune suppressive functions. Various human and murine Breg subtypes have been reported. While interleukin (IL)-10 intracellular staining remains the most reliable way to identify Breg cells, this technique hinders further essential functional studies. Recent findings suggest that CD9 is an effective surface marker of murine IL-10 competent Breg cells. However, the stability of CD9 and its relevance as a unique marker for human Breg cells, which have been widely characterized as CD24^hi^CD38^hi^, have not been investigated. Here, we demonstrate that CD9 expression is sensitive to in vitro B cell stimulations. CD9 expression could either be re-expressed or downregulated in purified CD9-negative B cells and CD9-positive B cells, respectively. We found no significant differences in the Breg differentiation capacity of the CD9-negative and CD9-positive B cells. Furthermore, CD9-positive B cells co-express CD40 and CD86, suggesting their nature as B cell activation or co-stimulatory molecules, rather than regulatory ones. Therefore, we report the relatively unstable CD9 as a distinct surface molecule, indicating the need for further research for a more reliable marker to purify human Breg cells.

## 1. Introduction

Regulatory B cells (Bregs) play a vital role in the maintenance of immune homeostasis and immunological tolerance. In contrast to the conventional B cells that carry out humoral responses, Breg cells negatively regulate the immune system by producing anti-inflammatory mediators, limiting T cell effector functions and differentiation as well as maintaining the activity of regulatory T cells (Tregs) [1]. The protective role of Breg cells in preventing widespread inflammation has been widely highlighted. Breg cell regulation against auto-antigens and pathogens has been described in various immune-mediated diseases, such as sepsis [2], allergic rhinitis [3], rheumatoid arthritis [4], inflammatory bowel diseases [5] and systemic lupus erythematosus [6,7].

Despite the critical roles of Breg cells in immune regulation, there are some discrepancies and inconsistencies concerning subset-defining markers. Breg cells often display highly phenotypic heterogeneity across different studies, including animal models and experimental settings [1]. There is a lack of subset-defining markers that can be utilized for an exclusive identification of human and murine Breg cells. To date, direct ex vivo isolation of Bregs is still not possible and the current most accepted identification method is by detecting intracellular expression of the immunosuppressive cytokine, interleukin (IL)-10, in B cells. This approach, however, limits a more detailed analysis on the ontogeny, function, and development of Breg cells. In mouse models, IL-10^+^ B cells have been assigned to multiple subsets, including splenic transitional 2 marginal zone precursor (T2-MZP), B10 CD5^+^CD1d^hi^, follicular (FO), plasma cells and TIM-1^+^B cells [8]. In humans, multiple studies have reported that CD24^hi^CD27^+^ and CD19^+^CD24^hi^CD38^hi^ are enriched in IL-10 and exhibit potent regulatory properties [6,9,10].

A recent study using a transcriptomic analysis reported that CD9 is a robust marker for murine Breg cells [11]. CD9 is a glycoprotein belonging to the tetraspanin family with diverse biological roles, including protein trafficking, cell adhesion and motility [12]. IL-10-expressing-B cells were predominantly found to be enriched within the CD9-positive population along with the upregulation of CD9 across different murine Breg subsets [11,12,13]. Furthermore, CD9 could be utilized to conveniently distinguish IL-10-competent B cell progenitors from conventional B cells [11,14]. Functional investigation additionally demonstrated that CD9-positive B cells efficiently curbed allergic responses and T cell-mediated inflammation in mice [14]. In complement, higher CD9 expression in human transitional CD24^hi^CD38^hi^ Breg cells and lower frequency of CD9 B cells in asthmatic patients have also been reported [13]. Mechanistic studies revealed that murine CD9 Breg cells exhibit IL-10-dependent regulatory roles in promoting apoptotic cell death in T cells [13]. However, the stability of the CD9 phenotype and its relevance in human Breg cells remain to be ascertained.

In the current work, we determined the phenotype stability of CD9 as a surface marker of human transitional Breg subsets. We first measured the frequency of CD9^+^ B cells in human tonsils under steady-state and stimulating conditions. Using fluorescence-activated cell sorting (FACS), we purified CD9-positive and CD9-negative B cells and monitored the changes in CD9 expression upon different culture conditions. The capacity for purified CD9-positive and CD9-negative B cells to differentiate into transitional cells and produce IL-10 was also investigated. Finally, we examined the co-expression of CD9 with other B cell-related markers.

## 2. Results

### 2.1. CD9 Is Expressed in Resting B Cells and Upregulated Upon Stimulation

**Figure 1:**
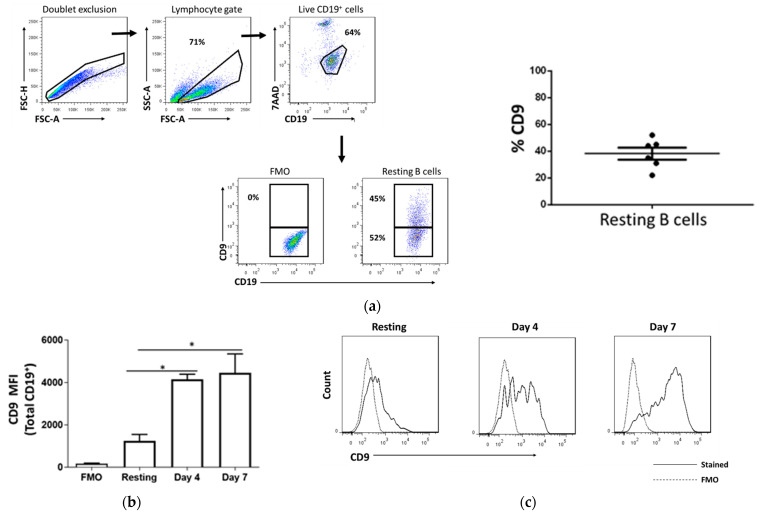
CD9 is expressed in resting tonsillar B cells and upregulated upon T cell-like (TCL) stimulation. B cells were purified from the tonsil using magnetic beads and analyzed for phenotypic CD9 expression. (**a**) Gating strategy for CD19^+^ B cells and its CD9 expression. (**b**) Purified naïve B cells were activated with TCL stimulation cocktail, CD40-agonist/anti-IgM/IL-2. On day 4 and 7, subsequent changes in CD9 expression were assessed and compared between the resting and stimulated B cells. (**c**) Representative histogram showing the mean fluorescence intensity (MFI) of CD9 in the resting and stimulated B cells. Error bars represent SEM; * *p* < 0.05. One-way ANOVA was performed to determine statistical significance. FMO, Fluorescence Minus One Control.

We first sought to examine the expression of CD9 in B cells using flow cytometry. B cells were purified using magnetic beads from tonsil cells, as detailed in the methods section. CD9 expression in B cells was analyzed with the gating strategy, as shown in Figure 1a. We found that in a steady-state condition, a proportion of resting B cells expressed CD9 (38 ± 10%) (Figure 1a). As B cells normally require antigenic stimulation to differentiate into effector or regulatory phenotypes, we then investigated the dynamism and stability of CD9 expression in response to B cell stimulation. Purified B cells were treated with a T cell-like (TCL) stimulation cocktail consisting of CD40-agonist, anti-IgM, and IL-2, which resemble signals from T helper cells, for 4 and 7 days. Although we did not detect any significant changes in the percentage of CD9-expressing B cells (data not shown), there was a substantial increase in the mean fluorescence intensity (MFI) of CD9 upon stimulation in a time-dependent manner (Figure 1b,c). This result suggests that while CD9 can be naturally expressed by resting B cells, its expression is not constant and may vary in different activation and development stages.

### 2.2. CD24^hi^CD38^hi^ B Cells Are Enriched within Activated Cd9^+^ B Cells

**Figure 2:**
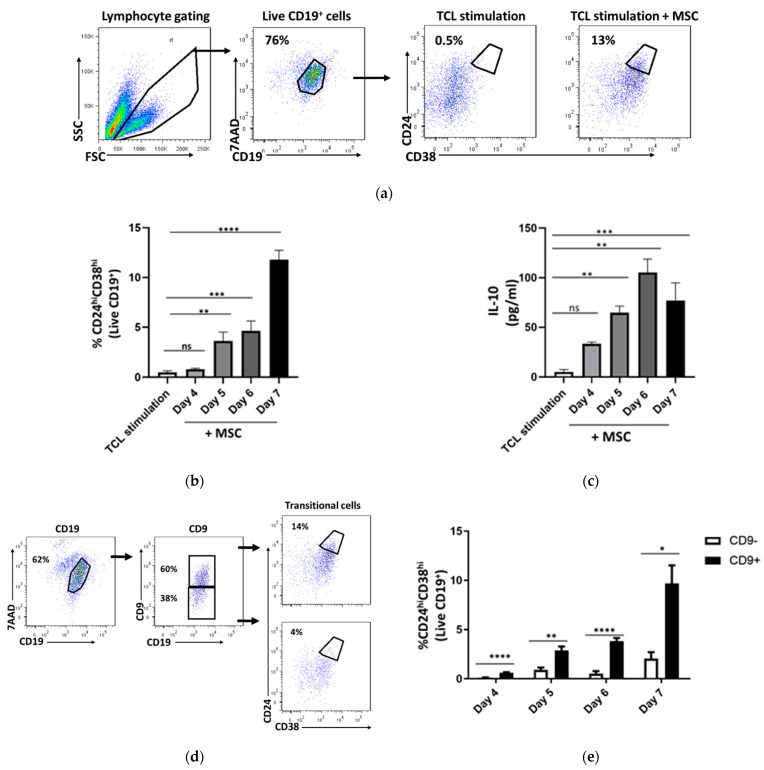
Transitional Breg cells are enriched within CD9-positive B cells. Naïve B cells were purified and activated with T-cell like (TCL) stimulation, in the presence of mesenchymal stem cells (MSC) for 7 days for the optimum induction of CD24^hi^CD38^hi^ transitional B cells. TCL stimulation without MSC serves as a control. (**a**) Flow cytometry gating strategy for analyzing transitional B cells. (**b**) Significant time-dependent increase of transitional B cells upon TCL stimulation with MSC. (**c**) Significant time-dependent increase of IL-10 in the culture supernatant upon TCL stimulation with MSC as measured by ELISA. (**d**) Representative gating strategy for the analysis of transitional B cells within CD9^−^ and CD9^+^ B cells. (**e**) Significant time-dependent increase of transitional B cell induction within CD9^+^ as compared to CD9^−^ B cells. Data show at least three independent experiments in each group. Error bars represent SEM; ns (non-significant) *p* > 0.05, * *p* < 0.05, ** *p* < 0.01, *** *p* < 0.001, **** *p* < 0.0001. Two-way ANOVA was performed to determine statistical significance.

CD19^+^CD24^hi^CD38^hi^ transitional Breg cells are the major IL-10-producing B cells and the most studied human Breg subset [6,15]. Thus, we aimed to verify the frequency of induced-CD24^hi^CD38^hi^ B cells in CD9^+^ B cells. For the induction of transitional cells, we used our established protocol, as previously published [16,17]. Briefly, B cells were purified and subjected to TCL stimulation as described above, with mesenchymal stem cells (MSC) at a 20:1 ratio (TCL+MSC) for 7 days. Stimulation without MSC served as a control. We adapted the gating strategy, as shown in Figure 2a, for analyzing transitional B cells. The transitional cells were defined as B cell populations with high expression of CD24 and CD38 (Supplementary Figure S1) as previously reported [9,10]. We first confirmed the potent capacity of this method in inducing transitional Breg cells at multiple time points. As expected, CD24^hi^CD38^hi^ B cells were significantly induced upon stimulation in the presence of MSC (day 7; TCL stimulation: 0.4 ± 0.4%, TCL + MSC: 11.78 ± 1.3%) (Figure 2a,b). In addition, the culture supernatant was collected at various times for the determination of IL-10 secretion using ELISA. In complement with the induction of transitional cells, the level of IL-10 was substantially increased upon TCL+MSC stimulation (day 7; TCL stimulation: 0.2 ± 0.2 pg/mL, TCL stimulation + MSC: 76.7 ± 40.1 pg/mL) (Figure 2c). Next, we assessed the frequency of transitional cells within the CD9^+^ and CD9^−^ B cell population. We found that the majority of transitional Breg cells were detected within CD9^+^ (day 7; CD9^+^: 9.6 ± 4%) as compared to CD9^−^ B cells (day 7; CD9^−^: 2.0 ± 1.4%) (Figure 2d,e). This demonstrates the enrichment of transitional B cells in the CD9^+^ population, as similarly reported by Brosseau et al. [13].

### 2.3. The Stability of CD9 as a Phenotypic Surface Marker

**Figure 3:**
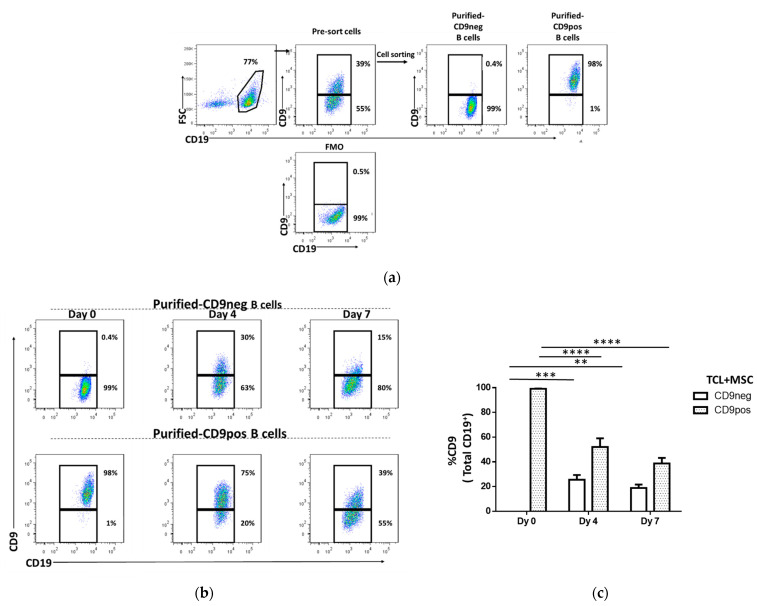
CD9 expression on B cells is transient. Tonsil cells were sorted into purified CD19^+^CD9^−^ (CD9neg) and CD19^+^CD9^+^ (CD9pos) B cells then cultured under the transitional cell-inducing condition with T-cell like (TCL) stimulation + MSC. At days 4 and 7, changes in the CD9 surface expression were analyzed with flow cytometry. (**a**) Representative dot plots showing the purity of CD9neg and CD9pos B cells. (**b**) Representative dot plots showing time-dependent changes in CD9 expression of the purified CD9neg and CD9pos B cells. (**c**) Significant gain and downregulation of CD9 expression in the purified CD9neg and CD9pos B cells, respectively. Data show at least three independent experiments in each group. ** *p* < 0.01, *** *p* < 0.001, **** *p* < 0.0001. Two-way ANOVA with multiple comparisons was performed to determine statistical significance.

Since most of the transitional Breg cells were predominantly detected within the CD9^+^ population, we thus monitored the stability of CD9 as a surface phenotype. Tonsil cells were labeled for CD19 and CD9, then samples were sorted into CD19^+^CD9^−^ (CD9neg) and CD19^+^CD9^+^ (CD9pos). After sorting, the purity of CD9neg B cells was >98% and CD9pos B cells was >96% (Figure 3a). Subsequently, sorted CD9neg and CD9pos B cells were induced into transitional cells using TCL stimulation in the presence of MSC, as described above. At days 4 and 7, CD9 expression was analyzed for time- and stimulation-dependent changes. Interestingly, we found that purified CD9neg B cells regained CD9 expression (17–40% at day 4 and 12–27% at day 7) (Figure 3b,c). On the other hand, CD9 expression was downregulated in the purified CD9pos B cells in which only 27–75% and 25–59% of CD9-expressing B cells were detected at days 3 and 7, respectively (Figure 3b,c). This observation suggests that CD9 expression can be induced and lost as well as influenced by culture conditions. This result suggests the plasticity of CD9 as a surface marker.

### 2.4. Transitional B Cell Induction Capacity on Purified CD9-Positive and CD9-Negative B Cells

**Figure 4:**
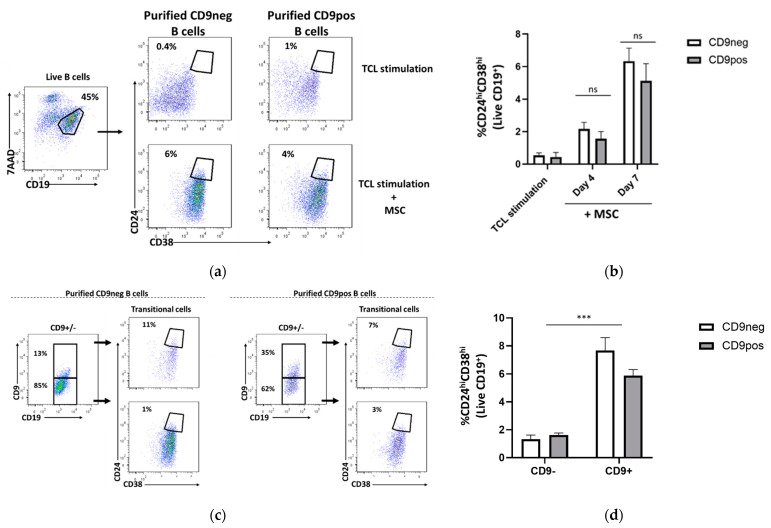
Transitional B cells can be induced in both purified CD9 negative- and positive-B cells at a similar level. Tonsil cells were sorted into purified CD9neg and CD9pos B cells then cultured under the transitional cell-inducing condition with T cell-like (TCL) stimulation + mesenchymal stem cells (MSC). At days 4 and 7, the frequency of CD24^hi^CD38^hi^ transitional B cells in both purified populations were examined. (**a**) Gating strategy and representative dot plots of transitional cell induction in purified samples. (**b**) No significant differences in the induction of transitional cells in both purified populations. Purified CD9neg and CD9pos B cells were re-stained for CD9 at day 7 of incubation and the frequency of CD24^hi^CD38^hi^ was analyzed within gated CD9^−^ and CD9^+^ cell in both purified populations. (**c**) Gating strategy and representative dot plots. (**d**) The frequency of CD24^hi^CD38^hi^ was significantly higher within gated CD9^+^ B cells as compared to CD9- in both purified populations. Data show at least three independent experiments in each group; ns (non-significant) *p* > 0.05, *** *p* < 0.001; two-way ANOVA was performed to determine statistical significance.

In murine experiments, sorted CD9^+^ B cells were shown to significantly express a higher level of IL-10 upon stimulation as compared CD9^−^ B cells [11]. This indicates that CD9^+^ B cells represent most mouse IL-10-competent B cell progenitors. Based on this finding, we then raise the question of whether CD9 serves as a surrogate marker of human Breg progenitor firstly by investigating the capacity of purified CD9pos and CD9neg to be induced into transitional B cells. Thus, following transitional cell induction with TCL+MSC, the frequency of CD24^hi^CD38^hi^ in the purified CD9pos and CD9neg B cells were assessed at days 4 and 7, using gating strategy as shown in Figure 4a. We found that CD9neg and CD9pos B cells can be transduced into transitional Breg cells at a similar level at day 4 (CD9neg: 2.1 ± 0.9%; CD9pos: 1.5 ± 1.2%) and day 7 (CD9neg: 6.3 ± 1.9%, CD9pos: 5.1 ± 2.9%) (Figure 4b). These data suggest that CD9pos and CD9neg B cells hold a similar transitional B cell induction potential. As we observed a homeostatic regulation of CD9 in both purified CD9neg and CD9pos B cells, the percentage of CD9^+^ and CD9^−^ B cells were re-evaluated at day 7 of incubation. The evaluation of CD9^+^ and CD9^−^ B cells among the purified CD9neg and CD9pos B cells was followed by the assessment of transitional cells within these subpopulations (Figure 4c). We found that the frequency of transitional B cells was statistically higher within CD9^+^ populations, in both purified CD9neg and CD9pos samples (Figure 4d). This finding indicates that B cells can differentiate into transitional cells regardless of their CD9 expression, though the frequency of transitional cells tend to be higher in CD9^+^ B cells.

### 2.5. IL-10 Production Capacity of Purified CD9-Positive and CD9-Negative B Cells

**Figure 5:**
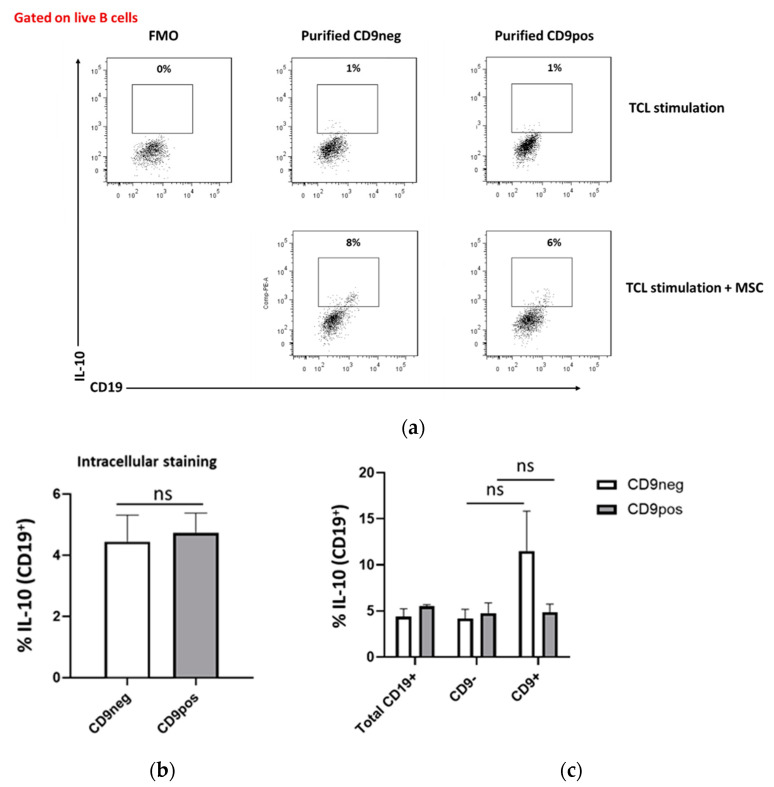
IL-10 expression can be induced in both purified CD9 negative- and positive-B cells. Tonsil cells were sorted into purified CD9neg and CD9pos B cells then cultured under the transitional cell-inducing condition with T cell-like stimulation + MSC. At day 7, the cells were re-stimulated with PMA/ionomycin/monensin and intracellular expression of IL-10 was analyzed. (**a**) Representative flow cytometry plots of IL-10 induction by MSC. (**b**) No significant differences in comparing IL-10 expression in the purified CD9neg and CD9pos B cell populations. (**c**) Purified CD9neg and CD9pos B cells were stained for CD9 at day 7 of incubation and IL-10 expression was analyzed within CD19^+^CD9^−^ and CD19^+^CD9^+^ gating. No significant differences in IL-10 compartmentalization within CD9^−^ and CD9^+^ of the purified cells. Data show at least three independent experiments in each group; ns (non-significant) *p* > 0.05. Unpaired *t*-test was performed to determine statistical significance. FMO, Fluorescence Minus One Control.

Next, we determined the capacity of both purified populations to express IL-10. As a potent immunosuppressive cytokine, IL-10 is a hallmark of Breg cells [18]. CD9neg and CD9pos B cells were re-stimulated with PMA, ionomycin, and monensin at day 7 for 4 h for the detection of intracellular IL-10. In comparison to the fluorescence minus one (FMO) control, both purified CD9neg and CD9pos B cells had a detectable IL-10 expression. However, we found no statistically significant differences when comparing the IL-10 expression in the two purified populations, suggesting the ability of both CD9neg and CD9pos B cells as IL-10 producers (Figure 5a,b). To further assess the compartmentalization of IL-10 expression, similarly, as described for Figure 4b, we gated on CD9^−^ and CD9^+^ B cell populations in the purified samples. IL-10 expression in the CD9^+^ subset of purified CD9neg B cells was higher compared to the other subsets, though it did not reach statistical significance (Figure 5c). These results suggest that IL-10 can be produced by B cells regardless of their CD9 expression, though CD9 could be linked to a de novo activation of resting B cells.

### 2.6. Expression of Other B Cell-Associated Markers in CD9^+^ and CD9^−^ B Cells

**Figure 6:**
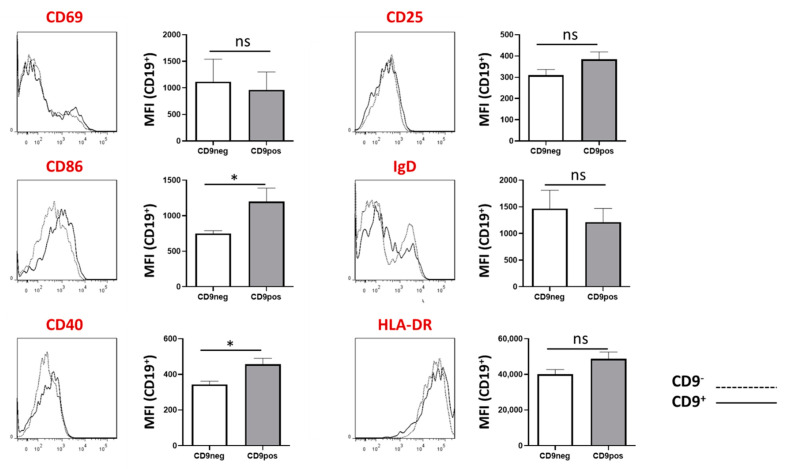
Expression of B cell markers in CD9^−^ and CD9^+^ B cell. Total tonsil cells were stained for CD19, CD9, CD69, CD86, CD40, CD25, IgD, and HLA-DR then subjected to flow cytometry analysis, of which live CD19^+^ was gated, followed by CD9^−^ and CD9^+^. Greater expression of CD86 and CD40 were detected within CD9^+^ B cells as compared to CD9^−^ B cells. * *p* < 0.05, ns (non-significant) *p* > 0.05. Solid line, CD9^+^ B cells; dotted line, CD9^−^ B cells. Student’s *t*-test (unpaired) was performed to determine statistical significance.

We further characterized CD9^+^ B cells by comparing the expression of B cell activation and developmental markers within CD9^+^ and CD9^−^ B cells using flow cytometry. Live CD19^+^ were gated into CD9^+^ and CD9^−^. We found that the expression of CD86 and CD40 but not CD69, CD25, IgD and HLA-DR were significantly higher in CD9^+^ B cells, as compared to CD9^−^ B cells (Figure 6). It is known that CD86 and CD40 are upregulated during B cell activation and are regarded as classical B cell activation markers. This finding suggests that CD9 may reflect the activation rather than the regulatory state of B cells. In addition, we also observed changes in other markers such as IgD, although the correlation was not significantly different. Further studies will be necessary to elucidate the precise roles of CD9 in B cells.

## 3. Discussion

The multiple yet overlapping Breg markers reported in numerous articles in the literature highlight the importance of identifying a unique surface marker that can represent the majority of Breg cells. So far, intracellular expression of IL-10 has been the common way to detect Breg cells. However, this method impedes important downstream studies. Recent reports have proposed CD9 as a robust marker for murine IL-10-competent B cells [11,13]. The finding was followed by several other reports demonstrating the potent ability of murine CD9^+^ B cells to suppress inflammation and T cell effector functions [12,13,14]. Thus, this study is designed to analyze the in vitro stability of CD9 as a phenotypic marker for human Breg cells, widely known as transitional CD24^hi^CD38^hi^ B cells.

We show that CD9^+^ B cells represent a proportion of tonsillar lymphocytes. T cell-like stimulation further upregulates CD9 expression in B cells. However, more importantly, B cells do not retain a stable expression of CD9. In purified CD9-positive and CD9-negative B cells, we observe that CD9 expression can be lost and regained. In response to Breg-inducing stimulation, there are no clear differences in the level of differentiation into transitional cells and IL-10 expression by CD9-positive and CD9-negative B cells. Furthermore, CD9 surface expression appears to be easily influenced by homeostatic regulation and may reflect the B cell activation status. Therefore, unlike its mouse counterpart, CD9 lacks the stability as a definitive marker for human Breg cells.

To our knowledge, the transient nature of CD9 as a surface marker has not yet been reported in other studies. Our findings suggest that CD9 is sensitive to cell culture conditions and subject to changes, which argues against what a unique surface marker should be and may not be useful for the future identification of naturally occurring human Breg cells.

Interestingly, transitional Breg cells in our study are often enriched within the CD9^+^ B cells, similar to what has been reported by other groups [12,13,14]. These data indicate that CD9 expression is a possible non-exclusive characteristic of differentiating B cells and play some other unknown active roles during B cell development, maturation, or activation. Indeed, several studies suggest that the unique pattern of CD9 expression is a property of different stages in B cell interaction, homing, migration, and survival [19,20]. As yet, the condition leading to CD9 upregulation in B cells is not yet understood. It is of interest to report here that we partly demonstrate that B cell activation, which closely mimics T cell-dependent stimulation, upregulates CD9 expression in B cells. Further research is needed to define the complete roles of CD9 in activated B cells.

Previous studies indicate that the majority of murine IL-10 expressing cells are originated from CD9^+^ B cells; however, we demonstrate here that CD9 is not a pre-requisite for B cells to differentiate into the transitional Breg phenotype and IL-10 producer [11]. Human B cells can differentiate into Breg cells, independent of their CD9 status. Therefore, CD9 is not a surrogate marker for Breg-competent cells in humans. There is still a significant gap in our knowledge of human and murine Breg cells. The majority of phenotypic and functional studies of Bregs have been extrapolated from mouse models; translating these findings in humans is proving to be challenging. While human and murine Bregs may share similar suppressive functions, differences in their phenotypes have been widely reported and discussed elsewhere [1,21]. As there is a minimal phenotypic overlap between human and murine Breg cells, it is theoretically challenging to attribute Breg phenotypes to a single marker. The instability of CD9 in human Breg cells reported in this study may owe to the interspecies variation and diversity of Breg, as well as CD9, biology. Indeed, CD9 is known as a multifunctional tetraspanin protein whose functions include cellular adhesion, migration, membrane fusion and signaling [12]. In addition, although IL-10 is regarded as the main distinguishing and undisputed feature of various Breg subsets, a recent report demonstrated the plasticity and transient nature of IL-10 expression in B cells [22]. Therefore, the lack of stability and ambiguity in Breg-related phenotypes may suggests that Bregs are not a specific subset with innate characteristics but their development is, rather, driven by the immune microenvironment as part of the adaptation to different stimuli [21,22,23].

While our investigation is primarily performed on CD24^hi^CD38^hi^ B cells derived from tonsils, the finding in this study is more likely to correspond to their splenic, peripheral blood and lymph node counterparts. Despite the heterogeneity of B cell subsets across different immune compartments, CD24^hi^CD38^hi^ B cells have been described to share common functional characteristics with major IL-10 producers and to exhibit comparable levels of immune regulatory activity [15]. Furthermore, relative to the circulating and splenic B cells, naïve tonsillar B cells are highly exposed to external environments and pathogens and, therefore, may better reflect B cell responses in the secondary lymphoid tissues during inflammation.

When analyzing the co-expression pattern of CD9 with other common B cells, we found that CD9^+^ B cells significantly display higher expression of CD40 and CD86, as compared to CD9^−^ B cells. CD40 and CD86 are well-known B cell activation markers and co-stimulatory molecules. Could CD9 reflect the general activation state, rather than the regulatory status, of B cells? This is not surprising. In fact, the majority of Breg-induction methods require the engagement of several B cell activation molecules, such as Toll-like receptors (TLRs), CD40, and B cell receptors (BCRs) [1,24]. More importantly, we observe that just a mere B cell stimulation in a non-Breg-inducing condition results in a higher expression of CD9. This further strengthens the hypothesis that CD9 represents B cell plasticity in response to the triggering of B cell receptors and CD40.

When interpreting these data, we should consider the potential suppressive role of CD9 within human B cells. Here, we do not exclude the possibility that CD9^+^ B cells may exhibit a higher suppressive capacity. Furthermore, it is also possible that CD9 is involved in regulating other phenotypical and functional properties of B cell functions. For instance, in this study, we have shown that CD9^+^ B cells exhibit activated B cell phenotypes. Moreover, as a tetraspanin, CD9 has been widely used as a marker for extracellular vesicles (EVs); therefore, it will be interesting to study whether CD9 has any effect on EV secretion in transitional and non-transitional B cells. Comprehensive investigation of the function of CD9 B cells will provide the opportunity to understand their precise roles more clearly.

Nevertheless, reliable surface markers for identification and isolation should be specifically and uniquely expressed. Beyond its purported marker for Breg cells, further investigation into the function of CD9^+^ and CD9^−^ B cells as well as correlations in different disease settings should be useful in providing more insight.

## 4. Materials and Methods

### 4.1. Mesenchymal Stem Cells

Mesenchymal stem cells (MSC) were derived from subcutaneous adipose tissues obtained from patients undergoing heart surgery in University Hospital Germans Trias i Pujol (HUGTiP). Written consent was obtained from all participants. The study protocol was performed according to the principles stated in the Declaration of Helsinki. Isolation of MSC from adipose tissues was performed as previously described [17,25]. MSC were cultured in αMEM (Sigma Aldrich, St. Louis, MO, USA) supplemented with 10% FBS (Lonza, Verviers, Belgium), penicillin (100 IU/mL, Cepa S.L., Madrid, Spain), streptomycin (100 mg/mL, Normon Laboratories S.A., Madrid, Spain) and 2 mM L-Glutamine (Sigma Aldrich).

### 4.2. B Cells Isolation

Tonsil cells were obtained from children undergoing routine tonsillectomy. Informed consent was obtained from all subjects through their legal tutors (HUGTiP). The study protocol followed the principles of the Declaration of Helsinki and was approved by the Clinical Research Ethics Committee of our institution (Comitè Ètic d’Investigació Clínica, Refs CEIC: PI-16-056 from 5 May 2016). A single-cell suspension was prepared from the tonsils by mechanical disruption with a scalpel and washed with sterile PBS. Subsequently, mononuclear cells were purified from total tonsil cells by differential centrifugation with Ficoll-Paque (GE Healthcare, Uppsala, Sweden) and stored in liquid nitrogen until use. Upon experiments, total mononuclear cells were thawed according to the standard protocol.

In some experiments, quiescent B cells were purified from thawed total mononuclear cells. Naïve B cells were negatively selected using CD43-magnetic beads (MACS, Miltenyi Biotec, Bergisch Gladbach, Germany) according to the manufacturer’s instruction (Supplementary Figure S2 for characterization of isolated tonsillar B cells). Purity was determined by flow cytometry (FACS Canto II, BD biosciences). Typically, cell suspensions consisted of >97% pure CD19^+^ B cells.

### 4.3. Cell Sorting

Thawed total mononuclear cells were washed with PBS and stained with CD19-BV510 (clone HIB19, BD Bioscience, San Diego, CA, USA) and CD9-FITC (clone HI9A, Biolegend, San Diego, CA, USA) in sterile FACS buffer, at room temperature. After 20 min incubation, cells were washed and resuspended in sorting buffer (PBS with 10% FBS, penicillin, and streptomycin). CD19^+^ cells were sorted into CD19^+^CD9^−^ (purified CD9neg) or CD19^+^CD9^+^ (purified CD9pos).

### 4.4. B Cell Stimulation and Culture

One-hundred thousand total B cells, or purified CD9-negative/positive B cells, were seeded in a flat-bottom 96-well plate and cultured in Iscove’s Modified Dulbecco’s Medium (IMDM, Lonza), supplemented with 10% FBS, 100 IU/mL penicillin, 100 mg/mL streptomycin and 2 mM L-Glutamine. To mimic T cell stimulation, a cell-activating cocktail containing 10 µg/mL F(ab)2 anti-IgM (Jackson, ImmunoResearch Laboratories, Inc., West Grove, PA, USA), 10^3^ IU IL-2 (Proleukin, Prometheus laboratories Inc., San Diego, CA, USA) and 2.5 µg/mL CD40 agonistic monoclonal antibody (BioXCell, Western Lebanon, NH, USA) was used. For the induction of transitional Breg cells, B cells were stimulated using the activation cocktail in the presence of 5000 MSC (T cell-like stimulation + MSC), as previously published by our group [16].

### 4.5. Detection of IL-10

After 7 days, B cells were harvested for analysis of IL-10 expression. For intracellular IL-10 expression, the B cells were restimulated with 50 ng/mL PMA (BD Biosciences), and 500 ng/mL ionomycin (BD Biosciences) for 4 h in the presence of 1× Monensin (BD Biosciences). The cells were stained with CD19-BV510 (clone HIB19) and IL-10-PE (Clone Jes3–9D7, Biolegend). Intracellular staining was performed with Intrastain kit (Dako, Denmark) according to the manufacturer’s instructions. The IL-10 level in the supernatant was quantified using IL-10-ELISA kits (U-CyTech, Utrecht, The Netherlands), according to manufacturer’s instructions. Cytokine standards were provided by the kit and a standard curve was prepared from 400 to 3.125 pg/mL. Samples and standards mixed with an antibody-coated 96-well plate were incubated for 2 h at 37 °C. The plates were washed and incubated with detection antibodies for 1 h.

### 4.6. Characterization of B Cell Surface Markers

For phenotyping of B cells, CD9^+^ and CD9^−^ B cells, the antibodies used were as follows: CD27-PE-Cy7 (clone 0323, Biolegend), CD38-PE (clone HB7, Biolegend), CD19-BV510 (clone HIB19, Biolegend), CD24-APC (clone SN3 A5-2H1D, eBioscience, San Diego, CA, USA), HLA-DR-PERCP (Clone L243, Biolegend), CD40-PE (Clone 5C3, BD Biosciences), CD86-PE (Clone 2331, BD Biosciences), CD25-PE/Cy5 (Clone M-A251, BD Biosciences), CD69-APC (Clone FN50, Biolegend), IgD-APC-Cy7 (Clone, IA6-2, Biolegend), IgM-FITC (Clone, SA-DA4, eBioscience), CD5-FITC (Clone, L17F12, eBioscience), CD10-APC (Clone, LT10, ImmunoTools, Friesoythe, Germany), CD20-PE-Dy647 (Clone, LT20, ImmunoTools) and CD21-FITC (Clone, LT21, ImmunoTools). Viability was assessed by 7AAD staining (BD Biosciences). All flow cytometric acquisition was performed with FACS Canto II, BD Biosciences. The data analysis was performed using FlowJo V7 (TreeStar Inc., Ashland, OR, USA). The gating strategy for the Bregs and single markers can be found in Supplementary Figure S1.

### 4.7. Statistical Analysis

Data are presented as mean ± standard error of the mean (SEM). The statistical significance was analyzed by ANOVA or Student’s *t*-test using GraphPad PRISM 7 software (GraphPad Software, San Diego, CA, USA). For all analyses, *p* values were indicated as * for *p* < 0.05; ** for *p* < 0.01; and *** for *p* < 0.001. The data show at least three independent experiments in each group.

## 5. Conclusions

The results of the present study demonstrated that, unlike what has been seen in the murine model, CD9 is not a stable Breg marker and should not be used for effective isolation of human Bregs from conventional B cells. These results highlight the need to further search for a more reliable phenotypic marker to identify Bregs in an effort to better understand its biology.

## Data Availability

All data generated during this study are included in this published article and its Supplementary Information.

## Supplementary Materials

Supplementary Materials can be found at https://www.mdpi.com/article/10.3390/ijms22094583/s1.
