# Supplementary material for: In Vitro Characterization of Human CD24hiCD38hi Regulatory B Cells Shows CD9 Is Not a Stable Breg Cell Marker
Source: Int J Mol Sci. 2021 Apr 27;22(9):4583. doi: 10.3390/ijms22094583 (PMC8123770; doi:10.3390/ijms22094583)
Supplement: Supplementary file 1 [file ijms-22-04583-s001.zip › ijms-1187219-supplementary.pdf]

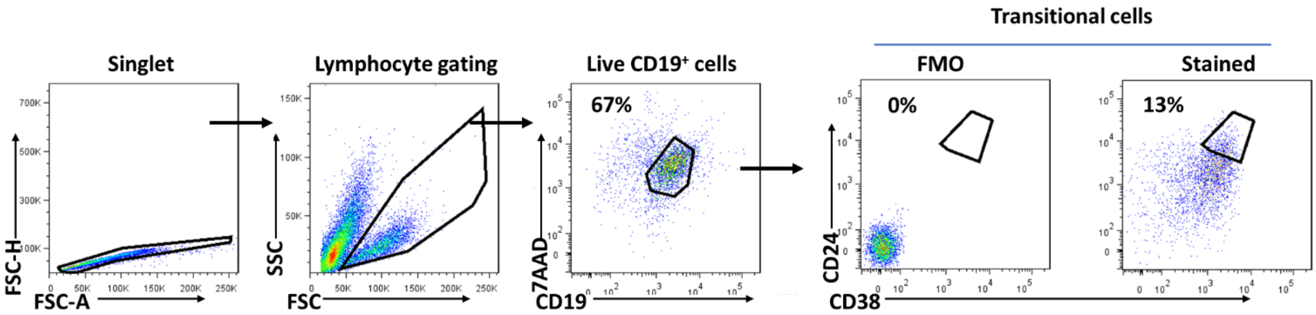

Supplementary Figure 1. Gating strategy for analysis of transitional $CD24^{hi}CD38^{hi}$ B cells. Singlets were identified using FSC-A versus FSC-H plot followed by FSC vs SSC plot for exclusion of debris and dead cells. Live $CD19^{+}$ cells were selected based on negative 7AAD staining. Subsequently, the gating of transitional cells was set on fluorescent minus one (FMO) control.

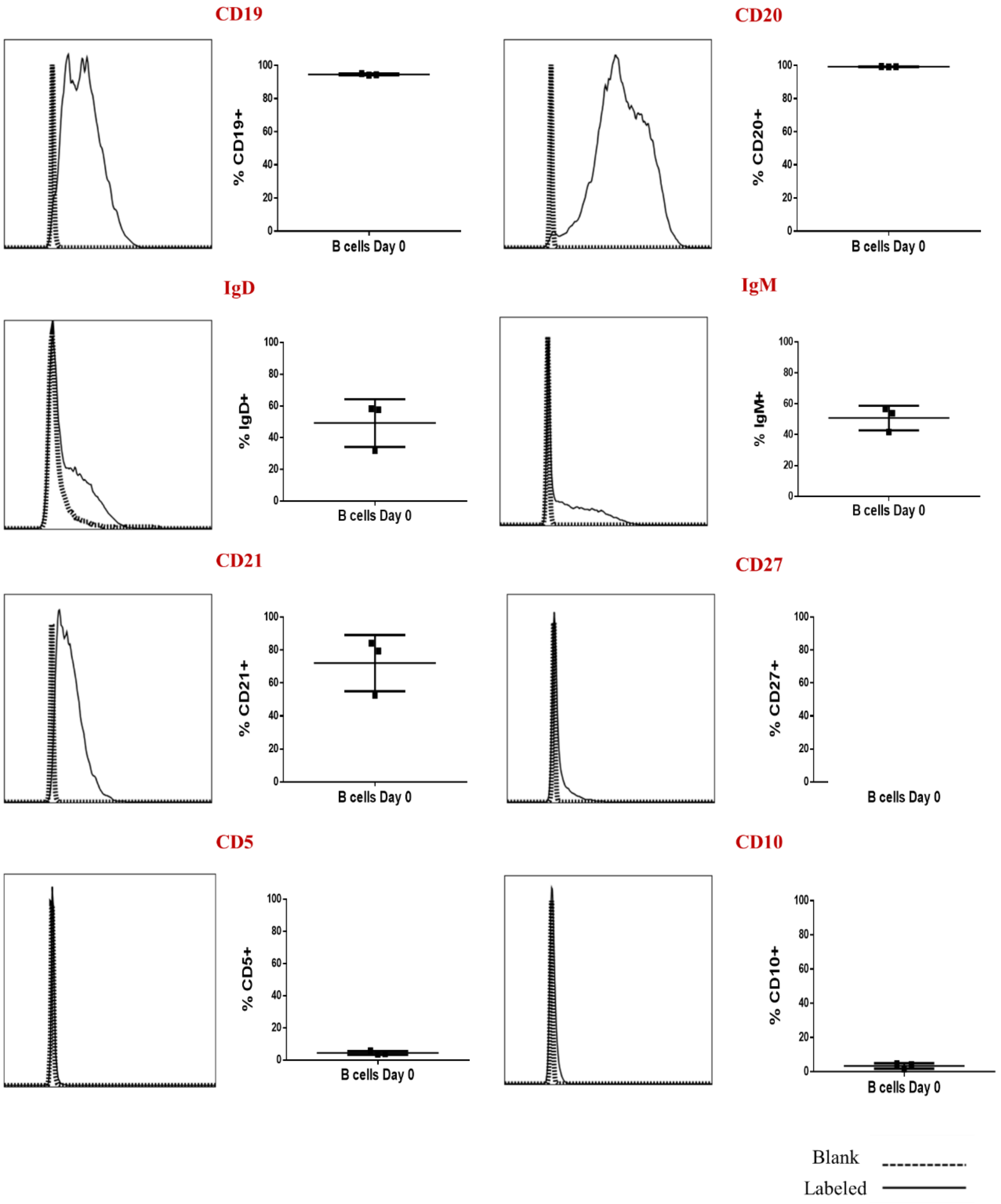

Supplementary Figure 2. Expression of B cell markers after CD43- isolation. Total tonsil cells were single stained for CD19, CD20, CD21, CD27, CD5, CD10, IgD and IgM then subjected to flow cytometry analysis. Solid line; Single Stain sample, Dotted line; Blank, no labelling with fluorochrome-conjugated, sample. Data shows B cells from 3 different tonsil donors.
